# Microplastic exposure across trophic levels: effects on the host–microbiota of freshwater organisms

**DOI:** 10.1186/s40793-022-00429-x

**Published:** 2022-07-06

**Authors:** Javier Edo Varg, David Outomuro, Warren Kunce, Lukas Kuehrer, Richard Svanbäck, Frank Johansson

**Affiliations:** 1 Department of Ecology and Genetics, Section of Animal Ecology, Evolutionary Biology Centre, Uppsala University, Norbyvägen 18D, 75236 Uppsala, Sweden; 2 Department of Aquatic Sciences and Assessment, Section for Ecology and Biodiversity, Swedish University of Agricultural Sciences, Undervisningsplan 7H, 756 51 Uppsala, Sweden; 3 Department of Biological Sciences, University of Pittsburgh, Pittsburgh, PA 15260 USA

**Keywords:** Anthropogenic stress, Dragonflies, Damselflies, *Daphnia*, Pesticide, Deltamethrin, Ecotoxicology

## Abstract

**Background:**

Microplastics are a pervasive pollutant widespread in the sea and freshwater from anthropogenic sources, and together with the presence of pesticides, they can have physical and chemical effects on aquatic organisms and on their microbiota. Few studies have explored the combined effects of microplastics and pesticides on the host–microbiome, and more importantly, the effects across multiple trophic levels. In this work, we studied the effects of exposure to microplastics and the pesticide deltamethrin on the diversity and abundance of the host–microbiome across a three-level food chain: daphnids–damselfly–dragonflies. Daphnids were the only organism exposed to 1 µm microplastic beads, and they were fed to damselfly larvae. Those damselfly larvae were exposed to deltamethrin and then fed to the dragonfly larvae. The microbiotas of the daphnids, damselflies, and dragonflies were analyzed.

**Results:**

Exposure to microplastics and deltamethrin had a direct effect on the microbiome of the species exposed to these pollutants. An indirect effect was also found since exposure to the pollutants at lower trophic levels showed carry over effects on the diversity and abundance of the microbiome on higher trophic levels, even though the organisms at these levels where not directly exposed to the pollutants. Moreover, the exposure to deltamethrin on the damselflies negatively affected their survival rate in the presence of the dragonfly predator, but no such effects were found on damselflies fed with daphnids that had been exposed to microplastics.

**Conclusions:**

Our study highlights the importance of evaluating ecotoxicological effects at the community level. Importantly, the indirect exposure to microplastics and pesticides through diet can potentially have bottom-up effects on the trophic webs.

**Supplementary Information:**

The online version contains supplementary material available at 10.1186/s40793-022-00429-x.

## Introduction

The large amount of microbes colonizing the host and covering all the mucosal surfaces such as digestive, respiratory tissues, and urogenital tracts is known as the host–microbiome. The gut microbiome has drawn most attention because of its relationship with diet and its importance in many aspects of the host’s health and well-being [1–4]. For example, most studies on wild animals have shown that diet in terms of prey species has a large effect on the microbial community composition [2, 5–7]. Other factors such as exposure to pollutants have been also shown to have an influence on host–microbiota [8–13]. Despite knowledge on the effects of prey consumption on the consumer’s host–microbiota, we know little about the potential carry-over effects of pollutants across trophic levels in predator–prey interactions.

Microplastics (MPs), defined as plastic polymer particles smaller than 1 µm, are pervasive emergent pollutants resulting from plastics that have been widely used in the last century, with a peak in production during the past decades [14–17]. MPs have become one of the largest wastes that are accumulated in the environment [14, 18]. Plastic debris and MPs in marine ecosystems are recognized as a global threat to marine organisms [19, 20]. In recent years, a lack of studies on plastics and MPs in freshwater ecosystems has been identified as a matter of priority [21]. Indeed, studies quantifying MPs, assessing MP exposure and MP uptake in freshwater organisms have been performed [22], demonstrating that MPs could have direct effects on the organisms, e.g., on life history-traits [22–24]. Moreover, the presence of ingested MPs in the gut imposes a threat as potential carriers of adsorbed hydrophobic organic chemicals or persistent organic pollutants that might be transferred to the organism [25, 26]. This might result in additive or synergic activities between MPs and other environmental pollutants such as pesticides, and MPs and pesticides might therefore have physical and chemical effects on the host–microbiota of aquatic organisms after ingestion [18, 27]. For example, Jin et al. [16] showed that MPs caused changes in the microbiota of mice, and these microbiome changes were suggested to affect metabolic disorders in the host. Nasuti et al. [12] showed that another stressor, the pyrethroid permethrin, reduced the abundance of several microbe groups in the guts of rats. However, few studies have focused on non-model organisms and on the combined effects of MPs and pesticides in the host and its microbiome.

Importantly, MPs and pesticides can have effects across trophic levels [28–31]. Changes in the nutrients and in the carbon source can modify the microbes in the environment [32, 33]. It has been shown that the microbiome is highly affected by food availability as well as habitat disturbance [34, 35], which potentially could result in bottom-up control of the microbes, i.e., affecting the microbiome of organisms higher up in the food chain [36–39]. Pervasive pollutants such as MPs could be colonized and used as a carbon source by some microorganism which in turn could interact with other stressors [40–42]. Hence, MPs colonized by microorganisms could interact with pesticides affecting bottom-up food web dynamics, but few studies are available on such interactions.

In this work, we examined the effects of exposure to MPs, with and without an additional stressor induced by sudden exposure to the pesticide deltamethrin (DMT), a pyrethroid. The pesticide DMT was chosen for examining effects on the microbiome because this pesticide is extensively applied as a pest control in agriculture, aquaculture, and forestry [43, 44]. DMT is known for its neurotoxic effects, acting mainly in the voltage-gated Na^+^ channels of the nervous system [43, 44], and in secondary targets involved in signal transduction [45, 46]. DMT has been shown to have negative effects on a variety of organisms including mammals and birds, and it is also highly toxic to aquatic organisms such as fish and aquatic invertebrates [43, 47]. Moreover, the effect of DMT in non-target organisms might be worsened due to the presence of other stressors, nutritional deficiencies, or other pollutants such as MPs [17]. Studies on the combined effects of MPs and DMT are rare [25, 48], and do not take into account the host–microbiome.

To examine the effects of MPs, and DMT on trophic levels, we studied the changes in the diversity and abundance of the host–microbiome in a three-level food chain: planktonic crustaceans (daphnids), predatory damselfly larvae, and top predatory dragonfly larvae. Our manipulation of pollutants occurred at the first (MPs) and second (DMT) food chain level. In addition, we estimated the survival of the damselfly larvae to the dragonfly top predator. We predicted: (1) an increase in the abundance of certain groups of microbes and decrease of diversity of the host–microbiome due to the exposure to MPs; MPs would behave as substrates for the microbial community, decreasing microbial diversity and increasing microbial abundance in functional digestion-related phyla such as Proteobacteria and Firmicutes [40, 49, 50]. (2) In the presence of DMT, we predicted a negative effect on the microbial diversity and abundance due to the pesticide bactericidal activity, affecting phyla such a Bacteroidetes that might be involved in gut barrier functions [13, 50]. (3) In the combined exposure to MPs and DMT, we predicted that the MPs might exert a sequestering effect on the pesticide by adsorption, resulting on lower effects on the diversity and abundance of the microbiome compared to separate effects of MPs or DMT alone. (4) We predicted a higher predation rate when the damselflies were exposed to MPs or DMT alone, due to a high accumulation of MPs in the body or intoxication by DMT. However, when the damselflies were exposed to both MPs and DMT, we hypothesized that the effect of the pesticide might be attenuated by the adsorption capacity of the MPs, resulting in lower predation rates than the exposure to MPs or DMT alone. (5) Finally, we predicted that microbiome effects occurring due to stressors at lower trophic levels might be carried over to higher trophic levels, even if the stressor is not physically transported to higher trophic levels.

## Material and methods

### Study species

The following species were used as the three-level food chain: the planktonic crustacean *Daphnia magna* Straus, 1820 as the resource level, larvae of the damselfly *Ischnura elegans* (Vander Linden 1820) as the intermediate predator, and larvae of the dragonfly *Aeshna cyanea* (Müller 1764) as the top predator. Daphnids are part of the diet of damselflies, and damselflies occur in the diet of dragonflies [51, 52]. All three species co-occur in waters in northern Europe (F. Johansson, unpublished).

### Experimental design

Two main experiments were performed to examine how the effects of exposure to pollutants at lower trophic levels affect the microbiome and how these effects are transferred to higher trophic levels. In the first experiment, daphnids were exposed to only MPs, only DMT, and a combination of both MPs and DMT. The control group was not exposed to either MPs or DMT. The daphnid microbiome was analyzed in this experiment.

**Fig. 1:**
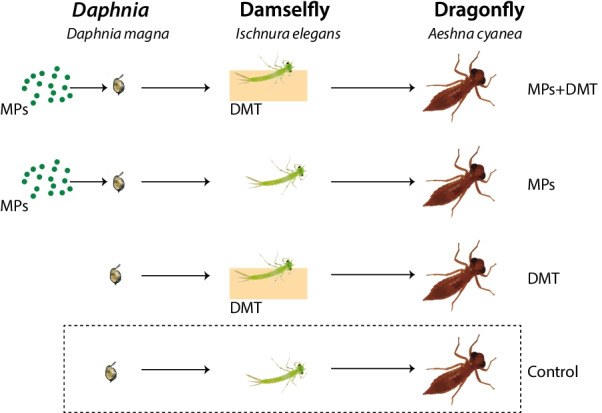
Overview of the experiment design showing the three-level food web. Half of the *Daphnia magna* were exposed to microplastics (MPs), and half were used as the control prey. The daphnids were then used to feed the damselfly *Ischnura elegans*. The damselflies were either exposed or not to the pesticide deltamethrin (DMT). Finally, the dragonfly *Aeschna cyanea* were fed with the damselflies*.* Neither of the three species were exposed to MPs or DMT in the Control group

In the second experiment, the effects of MPs and DMT were studied in the three trophic food chain (Fig. 1). Daphnids were divided into two groups, one exposed to MPs and one used as a control. These daphnids were then used to feed damselflies. Half of the damselflies were exposed to DMT simulating a sudden rainstorm causing a flush of pesticides potentially affecting freshwater organisms, resulting in the following four treatments: damselflies fed on control daphnids (Control group), damselflies fed on control daphnids, and exposed to DMT (DMT group), damselflies fed on daphnids exposed to MPs (MPs group), and damselflies exposed to DMT and fed on daphnids exposed to MPs (combined exposure group). Finally, the damselflies from the four treatments were offered to a dragonfly top predator. The microbiome of the damselflies and the dragonflies were analyzed, and the survival of the damselflies recorded. The dragonfly predator was not exposed to DMT because the aim of this experiment was to examine the sole effects of the transfer of MPs and DMT on the microbiome of the top trophic level.

### Pre-experimental setup

Laboratory cultured *D. magna* that had been kept in the laboratory for 5 years were used as the prey in the experiments. Prior to the start of the experiments, *Daphnia* were grown in a 70 L tank in aerated dechlorinated tap water. The temperature was 20 ± 1 °C, and the photoperiod was 16 h L: 8 h D. Before being used in the experiments, groups of 15 daphnids were transferred to 2 L plastic vessels with 1.2 L of dechlorinated tap water where they were fed *Raphidocelis subcapitata* algae*.* The algae were grown in modified Wright's cryptophyte (MWC) medium [53] in 1 L flasks stirred overnight.

Eggs of the damselfly *I. elegans* were obtained from 10 mated females collected with a butterfly net in Uppsala, Sweden (59.843715, 17.666730). The eggs were hatched in the laboratory, and after hatching larvae were randomly mixed and added to five plastic rearing containers (25 cm diameter, 12 cm height). The rearing containers were filled with 2 L of dechlorinated tap water and kept at 20 °C. Damselfly larvae were fed daily with brine shrimp *Artemia salina* (Linnaeus, 1758) and *D. magna*. All larvae were kept in the rearing containers until used in the experiments.

Larvae of the dragonfly *A. cyanea* were sampled using a hand net in a pond close to Uppsala, Sweden (59.852864, 17.472441). A total of 80 larvae were collected and transported to the laboratory in a 10 L plastic container with water and vegetation from the pond. In the laboratory, the dragonfly larvae were redistributed individually into small plastic containers (400 ml) with 150 mL of a mixture of tap water (dechlorinated tap water) and pond water. A branch of vegetation from the pond and a small stone were added to each container for habitat enrichment. Water temperature was kept at 20 °C. Dragonfly larvae were fed every other day with two *Chironomous riparius* (Meigen 1804) and with *D. magna*.

### Experimental setup

#### Concentration of MPs and DMT

In the environment, the highest reported waterborne concentration of MPs > 80 μm exceeds 100,000 particles m^−3^. However, due to the size of the MPs and the complexity of MP sampling, there is no comprehensive data of MPs < 333 μm [54, 55]. Due to constant fragmentation, the size of MPs would decrease and the particle concentration will increase. Therefore, a concentration and size of 7.8*10^5^ particles/mL (0.012 mg/mL) of 3 µm MPs spheres was used (Polybead Microspheres, CAS# 0,009,003,536, Polysciences, Inc.). Similar experiments used concentrations from 0.001 to 0.15 mg/mL, with a particle size between 100 nm and 10 µm [56–58].

Pyrethroid pesticides and DMT have a half-life that ranges from 25 to 72 days depending on the substrate, and they have been found in concentrations of 0.04–24 µg/L in agricultural areas, 0.1–6.0 µg/L in water bodies, and up to 100 µg/L in bottom sediments [44, 46, 59–61]. Other studies used a sub-lethal dose of DMT at concentrations of 0.25–15 µg/L [43, 46, 47]. The chosen concentration of DMT was therefore 0.2 µg/L of aerated DMT.

#### Daphnia

At the start of the experiment daphnids from the 2 L plastic vessels were moved to two 6 L glass containers, with approximately 2000 individuals per container. One container was exposed to MPs and the other container held only dechlorinated tap water (control). Intake of MPs was followed by visual inspection. However, a previous study [58] suggested that a complete egestion of 2 µm MPs in *Daphnia* does not occur within 24 h, meaning that in 48 h the animals will start a second round of MPs ingestion. The *Daphnia* were therefore exposed to MPs for 48 h. After this treatment, five daphnids from the MPs treatment and five from the control treatment were exposed to 0.2 µg/L aerated DMT solution individually for 24 h. This created four treatments: control, exposure to MPs, exposure to DMT, and exposure to both MPs and DMT. Four replicates per treatment (5 daphnids per vessel) were used. These *Daphnia* were subsequently stored at − 20 °C and used for microbiome analyses. The water was pooled per treatment and filtered with a 0.2 µm filter. The filters were stored at − 20 °C for further water microbiome analysis.

#### Damselflies

Before the experiment started, the damselfly larvae were placed individually in 50 mL glass vessels to be starved for 3 days. Thereafter the damselfly larvae were exposed to four treatments: control, exposure to MPs, exposure to DMT, and exposure to both MPs and DMT (Fig. 1). Each treatment consisted of 40 individuals. In the control treatment, damselfly larvae were fed five *Daphnia* from the *Daphnia* control treatment. In the MPs treatment, damselfly larvae were fed five *Daphnia* from the *Daphnia* MPs treatment. The DMT treatment consisted of damselfly exposed overnight (12 h) to 0.2 µg/L aerated DMT solution and fed five *Daphnia* from the *Daphnia* control treatment. Finally, the combined exposure treatment of MPs and DMT consisted of overnight exposed damselfly larvae to 0.2 µg/L aerated DMT solution, followed by feeding them with five *Daphnia* from the *Daphnia* MPs treatment. In all treatments (1 damselfly per vessel), each damselfly larva was allowed to feed on the five *Daphnia* for 4 h*.* All the damselfly larvae ate all the *Daphnia* provided. After this experiment, a minimum of three damselfly larvae from each treatment were stored at − 20 °C for further microbiome analysis.

#### Dragonflies

Dragonfly larvae were placed in individual plastic containers (9 cm height, 7 cm width, 7 cm length) and starved for 4 days prior to the start of the experiment. Each container was filled with 200 ml of dechlorinated tap water and had a small stone that served as a perch for the dragonfly. A second set of damselfly larvae were given the same four experimental treatments as described in the previous section (control, MPs, DMT, and combined MPs and DMT) and subsequently used for serving as prey for the dragonfly larvae (Fig. 1). Each damselfly was rinsed with aerated dechlorinated tap water 2 h before being used in this experiment. Hence, the dragonfly larvae were not exposed to the treatments per se, it was only the prey (damselfly larvae) that received these treatments. Within each individual dragonfly container, 3 damselfly larvae from the same treatment were added. Predation was noted upon 10, 20, 30, 60, 120, 180, 840, and 1440 min after adding the three damselfly larvae. Fifteen replicates were run for each treatment. After the 1440 min, the dragonfly larvae were stored at − 20 °C and later used for microbiome analysis.

### DNA extraction and library preparation

The whole microbiome was extracted and analyzed for all the *Daphnia* and damselfly after rinsing them with Milli Q water to avoid microbes from the water. The dragonfly larvae were dissected to be able to extract the whole gut microbiome. Larvae were first rinsed with Milli Q water, decapitated, and dissected with a sharp sterile blade to have access to the larvae gut. Using DNeasy Powersoil (Qiagen, No./ID: 12,888-10), DNA was extracted from the three species, and from the stored 0.2 µm filters used to filter the water that contained the daphnids. The manufacturer's protocol was followed with an additional incubation at 65 °C for 10 min after adding the C1 solution and additional 30 min of the bead homogenizer step*.* The 16S ribosomal RNA gene (16S rRNA) was amplified in a two-step PCR using primer pair 515F and 805R that flanks the hypervariable region V4. For the first step, PCRs were performed in triplicate using Phusion High-Fidelity DNA polymerase (Thermo Fisher Scientific, No./ID: F-530XL). Thirty cycles were performed following the Phusion polymerase protocol. Negative controls or blanks were run during DNA extraction and used as negative controls in the 16S rRNA PCR amplification to check for contamination. Triplicate PCR products of each sample were pooled and subsequently purified using AMPure XP magnetic beads (Beckman Coulter, No./ID: A63882). For the second step, Illumina adaptor sequences and barcodes were attached to the PCR primers to provide each sample with a unique identifier. Samples were then purified again using magnetic beads. An equal concentration of DNA from each sample was pooled and run through an agarose gel. Then, the 400–500 bp band was excised and purified using the QIAquick gel extraction kit (Qiagen, No./ID: 28,104). PCR products were sequenced on IlluminaMiSeq to obtain 250 bp paired-end reads at Science for Life Laboratory (SciLifeLab, Uppsala, Sweden).

### Sequencing data analysis

The *Daphnia*, damselfly, dragonfly, and the water microbiome (0.2 µm filtered water) amplicon sequence variant (ASV) tables were created using demultiplexed data from the SciLifeLab and following the DADA2 R pipeline 1.8 [62]. Taxonomy was assigned using SILVA database and singletons were filtered [63]. The alpha diversity and the most abundant phyla were calculated using the R packages lattice [64] and MASS [65]. A diversity analysis (phylogenetic, Shannon and Chao) was performed to obtain respectively ASV phylogenetic differences, ASV abundance and evenness, and ASV richness, using the R packages fossil [66], vegan (Oksanen et al. [72]), ape [67], and picante [68]. To test the effects of the exposure to MP and DMT on the microbiome, linear models were carried out using the diversity indexes as the response variable and the exposure to MPs and DMT (presence/absence) as fixed effects. Due to lack of normality, a permutation analysis was performed with 9999 permutations to confirm the robustness of the parametric model [69]. A post-hoc test was carried out for pairwise comparisons using the R packages FSA [70] and rcompanion [71].

The beta diversity was assessed using Permutational Multivariate Analysis of Variance (PERMANOVA) with normalized data and Bray–Curtis as a metric using the R package vegan [72]. The ASV distance matrix was used as a response variable, including the exposure to MP and DMT (presence/absence) as factors. To observe how the microbial communities cluster between treatments, a Principal Coordinates Analysis (PCoA) was performed. Multivariate Analyses of Variance (MANOVAs) were also run either using the relative abundances of the main six phyla or the six main genera as response variables, and MPs and DMT as fixed factors. To observe the effects of MPs and DMT in the relative abundance of each main phyla and genera, Generalized Linear Models (GLM) with a quasi-Poisson family were performed. Similarly, low abundant genera that ranked as main members of the main phyla and that constituted more than 1% of the total relative abundance were also evaluated. All the statistical analyses were executed in R statistical Computing Language 3.6.2 [73]. The phylogenetic tree and the taxonomy plots were created using Qiime 1.9.9 [74]. SILVA database was used as reference to make the tree [63].

### Predation analysis

The effects of the different exposure treatments on the damselfly survival against dragonfly predation at 10, 20, 30, 60, 120, 180, 840, and 1440 min were analyzed using Generalized Linear Mixed Models (GLMM) with multivariate normal random effects, using Penalized Quasi-Likelihood. The response variable was entered as counts per vessel of living and predated damselfly larvae for each time period. Time was entered as a covariate, and the exposures (presence/absence) to MP and DMT were entered as fixed factors. The dragonfly ID was entered as a random effect. Finally, to account for repeated measurements an autocorrelation structure of order one was entered, with time as a continuous covariate and dragonfly ID as a grouping effect. The model was performed using the R packages MASS [65] and nlme [75]. A post-hoc test was performed to observe differences between treatments using the R package emmeans [76].

## Results

### Alpha and beta diversity of the host–microbiome

**Fig. 2:**
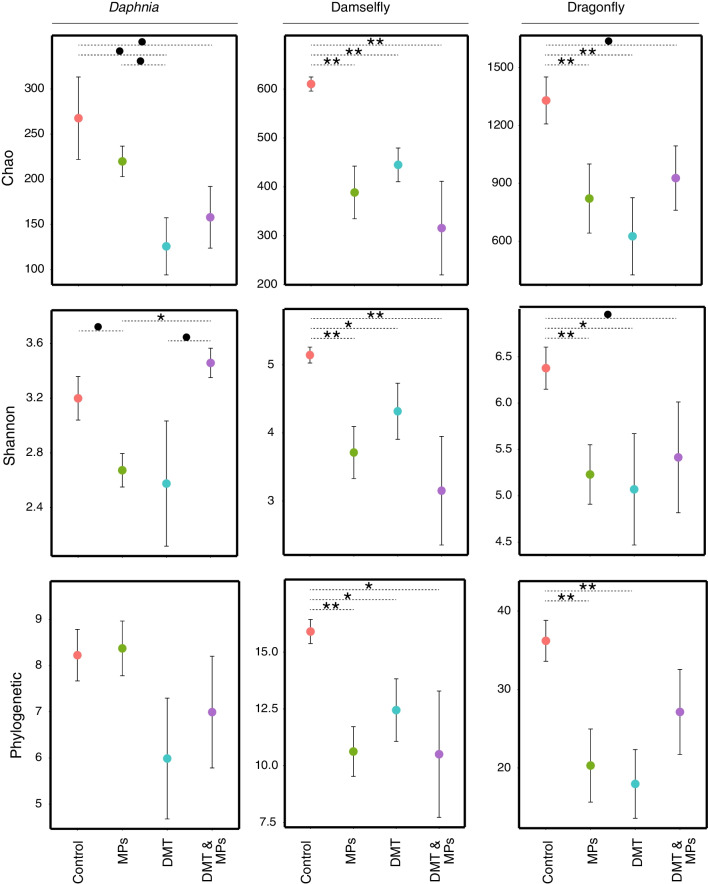
Diversity indexes Chao, Shannon and Phylogenic for the host–microbiome of *Daphnia*, the damselfly larvae and the dragonfly larvae. The aquatic invertebrates were exposed to microplastics (MPs), deltamethrin (DMT) or a combination of both. The animals not exposed to either MPD or DMT were the control group. Significant differences between treatments were tested using post-hoc Tukey tests (***: *p*-value < 0.001; **: 0.001 < *p*-value < 0.01; *: *p*-value < 0.05; •: 0.05 < *p*-value < 0.09)

In the daphnid microbiome, the Chao diversity index was negatively affected by the exposure to DMT, i.e., a decrease of diversity (Table 1, Fig. 2). For the Shannon diversity index, the combined exposure to MPs and DMT had a significant positive effect on the microbial diversity (Table 1, Fig. 2). The Phylogenetic diversity index only showed a marginally non-significant effect by the exposure to DMT (Table 1, Fig. 2). We refer to marginally non-significant effects in instances where the *p*-value was between 0.05 and 0.09. The post hoc analyses showed that for the Shannon index, the MPs treatment was significantly different compared to the combination of MPs and DMT (Fig. 2). In addition, there were some marginal non-significant differences between treatments in the Shannon and Chao indexes (Fig. 2).

**Table 1:** Effects of exposure to microplastics (MPs) and deltamethrin (DMT) on the host microbiome of *Daphnia*, damselfly larvae and dragonfly larvae

Organism	Variable	MPs	DMT	MPs x DMT
*Daphnia*	*Chao*			
	F_3,13_	0.623	6.940	1.135
	*p*-value	0.444	**0.021**	0.306
	*Shannon*			
	F_3,13_	0.523	0.482	12.057
	*p*-value	0.482	0.5	**0.004**
	*Phylogenetic*			
	F_3,13_	0.053	3.347	0.194
	*p*-value	0.821	**0.09**	0.666
	*Permanova*			
	F_3,13_	0.604	1.356	2.729
	*p*-value	0.696	0.248	**0.027**
Damselfly	*Chao*			
	*F*_3,20_	20.281	5.344	0.787
	*p*-value	** < 0.001**	**0.032**	0.386
	*Shannon*			
	F_3,20_	16.244	3.059	0.111
	*p*-value	** < 0.001**	0.101	0.743
	*Phylogenetic*			
	*F*_3,20_	16.226	2.02	1.67
	*p*-value	** < 0.001**	0.171	0.211
	*Permanova*			
	F_3,20_	6.876	1.815	0.888
	*p*-value	**0.005**	0.159	0.403
Dragonfly	*Chao*			
	F_3,32_	2.958	2.639	4.569
	*p*-value	**0.096**	0.116	**0.042**
	*Shannon*			
	F_3,32_	4.308	2.061	3.436
	*p*-value	**0.045**	0.156	**0.071**
	*Phylogenetic*			
	F_3,32_	4.666	1.621	7.136
	*p*-value	**0.041**	0.210	**0.013**
	*Permanova*			
	F_3,32_	2.626	0.663	2.467
	*p*-value	**0.016**	0.749	**0.020**

The host microbiome was studied as Alpha (Chao, Shannon, Phylogenetic) and Beta diversity (Permanova). Significant and marginally non-significant p-values are highlighted in bold

The three alpha diversity indexes of the damselfly host–microbiome were all negatively impacted by the exposure to MPs (Table 1, Fig. 2). There was also a negative significant effect by the exposure to DMT on the Chao index (Table 1). Post hoc contrasts showed that for all the alpha diversity indexes, the control was significantly higher compared to other treatments (Fig. 2).

For the diversity of the dragonfly microbiome, the exposure to MPs negatively affected the Shannon and phylogenetic indexes, whereas the effects on the Chao index were marginally non-significant (Table 1, Fig. 2). Moreover, the combined exposure of MPs and DMT negatively affected the Chao and phylogenetic indexes whereas there was only a marginally non-significant effect on the Shannon index (Table 1). Post hoc contrasts showed that the control had significant or marginally non-significant higher diversity than the other treatments, except for the phylogenetic diversity index where the control had significantly higher diversity than the exposure to MPs and DMT alone (Fig. 2).

**Fig. 3:**
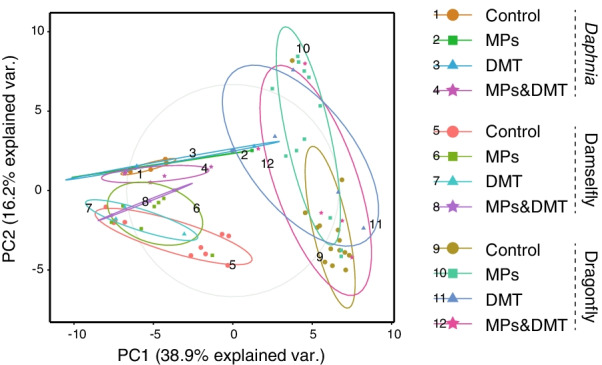
Principal Coordinates Analysis showing the microbial composition clusters of the microbiome of *Daphnia*, the damselfly and the dragonfly. The microbial composition in each species is coded following the exposure treatment to microplastics (MPs), the pesticide deltamethrin (DMT), a combination of both, and without either of them (Control)

There were distinct clusters in the host–microbiome for each host species, i.e., in the daphnids, damselfly larvae, and dragonfly larvae (Fig. 3). The beta diversity of the daphnids was significantly affected by the combined exposure to MPs and DMT (Table 1: Permanova). In the case of the damselfly larvae, the beta diversity was affected by the exposure to MPs (Table 1: Permanova). Finally, the beta diversity of the dragonfly larvae was affected by the exposure to MPs and the combined exposure to MPs and DMT (Table 1: Permanova) (Additional file 1).

### Main phyla and genera of the microbiome

**Fig. 4:**
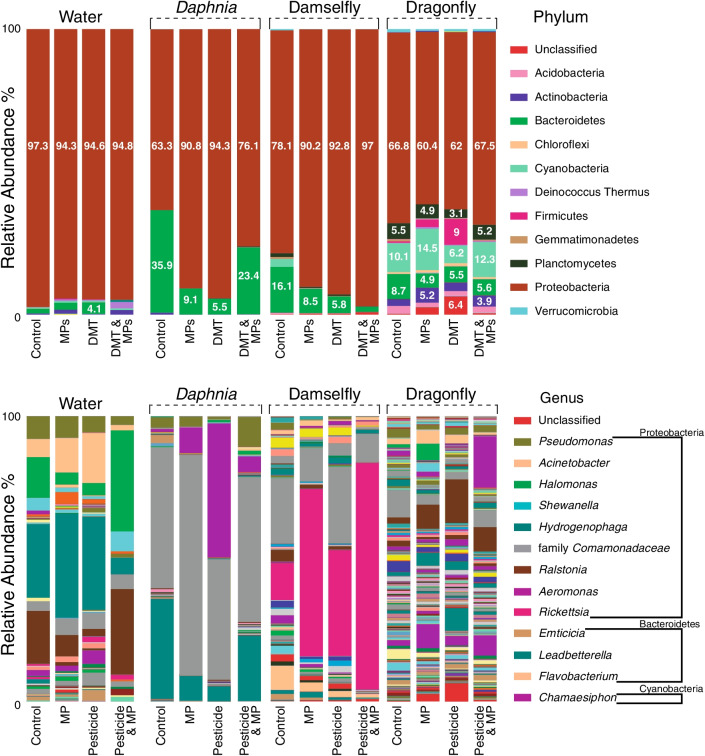
Relative abundance of the microbiome of *Daphnia*, the damselfly and the dragonfly, including the relative abundance of the water microbiota extracted from the filters at phylum and genus level. The exposure treatments were: microplastics (MPs), the pesticide deltamethrin (DMT), a combination of MPs and DMT, and the Control group (no exposure to either MPs or DMT)

The six main phyla and genera were analyzed for each host species (Additional file 2: Tables S1–S9). Four of these phyla were shared between the daphnids, the damselflies, and the dragonflies and the most abundant taxa were Proteobacteria, Bacteroidetes, Cyanobacteria, and Planctomycetes. There were no shared genera between the daphnids, the damselflies, and the dragonflies. However, they shared members from the family *Comamonadaceae*. It was not possible to assign taxonomy at the genus level for members of that family. The microbiomes of all the hosts were dominated by the phylum Proteobacteria, and this was also the case for the water samples. Consequently, the most abundant genera belong to the phylum Proteobacteria (Fig. 4). The relative in abundance of Gammaproteobacteria, Betaproteobacteria, Alphaproteobacteria and other taxa at class level can be observed in Additional file 2: Fig. S1.

In Daphnia, the MANOVA showed no significant changes in the abundance of the main microbe phyla when exposed to MPs and/or DMT (Additional file 2: Table S1). However, there were significant effects in the relative abundance in the individual phyla: increase of Proteobacteria, decrease of Bacteroidetes, and decrease of Actinobacteria by the exposure to MPs, DMT, and their combination; Planctomycetes increased by the exposure to DMT (Additional file 2: Table S1, Fig. 4). The post hoc contrasts on the univariate relative abundances showed no significant differences between treatments (Additional file 2: Table S1). Similarly, at the genus level, the MANOVA showed no significant changes in the abundance of the main genera (Additional file 2: Table S2). At the genus level, the relative abundance of *Leadbetterella* [Relative abundances: Control: 35.1%, DMT:5.3%, MPs:8.7%, DMT and MPS: 22.9%] and *Limnobacter* [Relative abundances: Control: 1%, DMT:0.1%, MPs: < 0.01%, DMT and MPS: 0.4%] decreased significantly due to the MPs. *Leadbetterella* was also significantly affected by DMT exposure and the combined exposure to MPs and DMT (Additional file 2: Table S2). The effects of MPs and DMT exposure in the main, but low abundant, genera of the phyla Bacteroidetes, Cyanobacteria, and Planctomycetes can be observed in Additional file 2: Table S3.

In the damselflies, the MANOVA showed marginal non-significant effects due to the exposure to MPs in the main phyla (Additional file 2: Table S4). On the other hand, the MANOVA showed significant effects on the abundance of the main genera due to the MPs exposure (Additional file 2: Table S5). The post-hoc tests showed that there were significant differences between the control and the MPs exposure and the combined exposure to MPs and DMT (*p* < 0.05). For the individual phyla, MPs affected increased the relative abundance of Proteobacteria, while decreasing the relative abundances of Cyanobacteria, Planctomycetes and Gemmatimonadetes (Additional file 2: Table S4, Fig. 4). In addition, DMT also decreased Cyanobacteria (Additional file 2: Table S4, Fig. 4). MPs also affected the relative abundance by increasing the unclassified Phyla (Additional file 2: Table S4, Fig. 4). The post hoc contrasts on the univariate relative abundances showed significant differences for Proteobacteria between the control and the combination of MPs and DMT, as well as for the unclassified taxa between the control and MPs, and the control and the combination of MPs and DMT (Additional file 2: Table S6). The main but low abundant genera showed a decrease in the relative abundance of the genus *Leptolyngbya* [Relative abundances: Control: 2.8%, DMT: < 0.01%, MPs: 0.1%, DMT and MPS: < 0.01%] due to MPs and DMT, a decrease in the family *Sphingomonadaceae* [Relative abundances: Control: 4%, DMT:1%, MPs: 1.3%, DMT and MPS: < 0.01%], and an increase in the unclassified taxa due to MPs [Relative abundances: Control: 0.1%, DMT:0.4%, MPs: 0.4%, DMT and MPS: < 0.9%] (Additional file 2: Table S7).

In the dragonflies, the MANOVA showed that the exposure to MPs significantly affected the abundance of the six main phyla (Additional file 2: Table S8). In the main genera, the MANOVA showed significant effects due to the exposure to MPs and a marginally non-significant effect due to the combined exposure to MPs and DMT (Additional file 2: Table S9). The univariate analysis showed that the MPs treatment decreased the relative abundance of Bacteroidetes and Acidobacteria, and increased Actinobacteria (Additional file 2: Table S8, Fig. 4). DMT and the two-way interaction of MPs and DMT significantly affected Planctomycetes (Additional file 2: Table S8, Fig. 4). In the main genera, *Rhodobacter* [Relative abundances: Control: 3.8%, DMT: 2.5%, MPs: 1.7%, DMT and MPS: 1.9%] decreased in relative abundance while the relative abundance of *Acinetobacter* [Relative abundances: Control: 2%, DMT: 2.8%, MPs: 4.5%, DMT and MPS: 0.8%] increased due to MPs (Additional file 2: Table S9). The relative abundance of unclassified taxa increased significantly due to MPs and DMT exposure but decreased due to the combined exposure to MPs and DMT (Additional file 2: Table S10). The post hoc contrasts on the univariate relative abundances showed significant differences for Bacteroidetes and Actinobacteria between the control and the MPs treatments (Additional file 2: Table S6). Class taxa level is shown in Additional file 2: Fig. S1.

### Damselfly survival

**Fig. 5:**
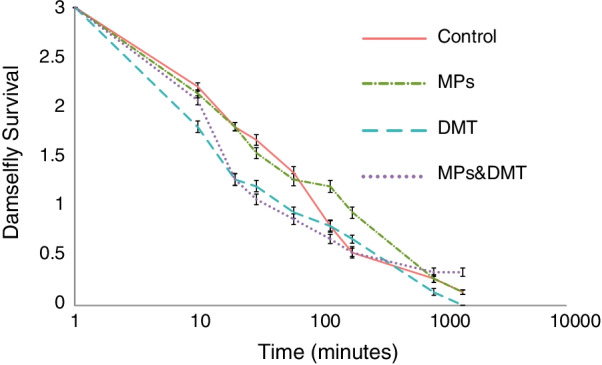
Damselfly survival from dragonfly predation over time when the damselfly larvae were exposed to microplastics (MPs), the pesticide deltamethrin (DMT), to both MPs and DMT (MPS&DMT) or to none of these stressors (Control)

The damselfly survival decreased across time and was negatively affected by the exposure to DMT alone, but not by the exposure to MPs or the combined exposure (Additional file 2: Table S11, Fig. 5). There were no significant two-way or three-way interaction effects between MPs, DMT, and time (Additional file 2: Table S11).

## Discussion

The main aim of this study was to investigate how the exposure to pollutants at lower trophic levels affects the microbiome at higher trophic levels, as well as how the pollutants affect predation on the intermediate level by the top predator. The microbiome was affected by the pollutants in the daphnids, the damselflies, and the dragonflies. The results thus suggest that the microbiome effects were transferred from lower to higher trophic levels, showing effects and changes on the microbial composition at the top trophic level. In addition, deltamethrin exposure on the damselflies affected their survival rate in the presence of the predator, but no such effects were found from microplastics exposure.

### *Effects on the* Daphnia *microbiome*

The diversity of the daphnid microbiome decreased in treatments with DMT and the combination of DMT and MPs. When inspecting the most abundant phyla, there were effects by the exposure to MPs, DMT, and their combination on Proteobacteria, Bacteroidetes, and Actinobacteria. Previous studies have shown that the microbiome of *D. magna* is primarily colonized by Proteobacteria and Bacteroidetes [77] and this is consistent with the most abundant phyla found in *D. magna* in the present study. In vertebrates, a decrease of Bacteroidetes might be related to abnormal intestinal permeability and pro-obesity phenotype [8, 13, 78]. The observed significant effect of Proteobacteria and Bacteroidetes in our experiment by the exposure to MPs and DMT supports our first and second predictions. These effects might have an impact on the health of *D. magna.* In support of this, previous studies on *D. magna* exposed to MPs or pesticides found effects on the daphnid fitness including growth, reproduction, feeding ability, and mobility [24, 48, 56, 79, 80]. In the case of the exposure to MPs, the effects on fitness seem to vary depending on the size and the material of the MPs [24, 25, 48, 56, 58, 79]. Moreover, the combined exposure to MPs and pesticides can have diverse effects. For example, the MPs can enhance the negative effects of the pesticide in *D. magna* [48]. The MPs can also provide more available areas for the chemical to bind, changing the concentration of the pesticide in the environment and therefore decreasing the toxic effect [25].

### Effects on the damselfly microbiome

The microbial diversity was also affected in the damselflies. There was a clear negative effect on alpha and beta diversities caused by the ingestion of daphnids exposed to MPs. This effect in the damselfly microbiome was indirect because the damselflies were not exposed directly to the MPs. The few studies available on how MPs might affect the microbiome of aquatic organisms have shown that the microbiome can be highly affected because the microorganisms can colonize the MPs [40, 42]. This colonization of MPs is known to cause changes on the microbial composition in vertebrates such as zebrafish [81] and in invertebrates such as Collembola [82]. However, our study is the first to show indirect effects of MPs on the microbiome via transfer through the food chain. These indirect effects might be widespread and require more attention in future studies.

Our second prediction was that the pesticide should affect abundance and diversity of the microbiome negatively. In general, we found support for this, but some phyla increased in abundance. The direct exposure to DMT caused a negative effect in the Chao diversity of the damselfly microbiome. Moreover, the exposure to MPs and DMT alone had some effects on the relative abundance of some of the main phyla of the damselfly microbiome. Such effects have rarely been studied in invertebrates, but severe changes of the microbiome in vertebrates have been found when they were exposed to stressors [4, 83, 84], and the change might have a large effect on the host health. For example, an increase of Proteobacteria might influence inflammation, lipid metabolism disorder, increase the susceptibility to infections, generate motor disabilities and gut diseases [12, 13]. We predicted an increase in abundance of Proteobacterio at the MP exposure and we found some support for this. We found that MPs increased relative abundance of Proteobacteria. It has been reported that some members of the phylum Proteobacteria showed higher abundance in mucosal compartments as mucosa-associated microbiota [49]. However, an increase in the abundance of Proteobacteria compared to the control was also observed in the DMT and the combined exposure treatments. In the combined treatment, the relative abundance of all the other phyla represented less than 2% of the total relative abundance.

### Effects on the dragonfly microbiome

The diversity of the host–microbiome of the top predator, the dragonfly, decreased by the exposure to MPs alone and by the combined exposure to MPs and DMT. Moreover, there were also changes in the relative abundance of the main microbiome phyla due to the exposure to MPs and DMT, either alone or in combination. It is very important to note that the MPs treatment occurred two trophic levels below (daphnids were directly exposed to MPs), and the DMT treatment occurred one trophic level below (damselflies were directly exposed to DMT). These results show that stressors at lower levels can affect the host–microbiome of organisms at higher trophic levels, including top predators that are not exposed directly to these stressors. The decrease in the diversity of the dragonfly microbiome is mirrored at the lower trophic levels. Interestingly, it was only in dragonflies that the differences in the total abundance of the main phyla were significant due to the exposure to MPs (Table 1: Manova) indicating that the effect of MPs is transferred through the food chain and maybe the MPs themselves [28, 29, 31]. Previous studies have suggested that the transfer of MPs through food chains might indicate a new threat due to MP contamination of soils [85–87]. This threat might be even higher in metamorphic organisms that could translocate the MPs from aquatic to terrestrial environments [88]. Finally, the combined treatment of MPs and DMT showed significant effects in the overall microbiome diversity, but small effects in the relative abundance of the main phyla. We argue that this effect could be due to the adsorption and absorption effects that the MPs might have [17, 18]. Hence we found some support for our third prediction.

### Comparing effects across trophic levels

Comparing the three trophic levels suggests that MPs affected the diversity of the organisms on the higher trophic levels, the damselflies and the dragonflies. For example, MPs significantly affected Bacteroidetes in daphnids and dragonflies, and Proteobacteria in daphnids and damselflies. Interestingly, MPs and DMT affected Cyanobacteria only in the damselflies. This is worrisome since it has been found that Cyanobacteria could be harmful for small invertebrates such as zooplankton and aquatic vertebrates due to toxin production [89, 90]. Proteobacteria, Bacteroidetes and Cyanobacteria are members of the six more abundant phyla in all the organisms of the trophic chain. The close similarity between *Daphnia* microbiome and damselfly microbiome compared to the dragonfly microbiome can be observed in Fig. 3. Furthermore, the changes in relative abundance at the genus level are consistent with the changes in relative abundance at the phylum level in the *Daphnia*, the damselflies, and the dragonflies.

### Predation experiment: damselfly larvae survival

In contrast to our fourth prediction, the damselfly larvae were indirectly exposed to MPs and this did not cause any differences in survival. Thus, even though the MPs affected the microbiome of the damselflies, the microbiome change seemed to have no effect on predator avoidance by the damselflies. It has been previously shown that MPs could have no effect on survival [23]. For a complete mechanistic understanding of survival in organisms exposed to MPs, future experiments should also inspect foraging ability, escape behaviors, prey mobility rate and life cycle effects across the entire life span in response to predators. In contrast to the MPs exposure, the damselflies exposed to DMT showed higher mortality. One reason for this could be that the toxicity of DMT affects the damselfly behavior. For example, Janssens and Stoks [91] showed that pesticide exposure and predation risk, and their interaction, had an effect on the behavior of a damselfly larvae in response to upregulating processes linked to detoxification. Similarly, a previous study showed that MPs in combination with a pesticide affected the swimming patterns and speed of a ciliate, *Favella* sp. [28]. This agrees with the well-known interference that DMT and other pesticides have in the voltage-gated Na^+^ channel of the nervous system [43, 44]. Interestingly, and as we predicted, the interaction between MPs and DMT showed no effect on predation risk. This might be due to the binding effect that the MPs have, consequently reducing larval exposure to the pesticide [17, 18].

### Food chain effect

Our results clearly showed that the effect of the exposure to pollutants at lower trophic levels affects the microbiome of organisms at higher trophic levels, despite the fact that the higher trophic levels were not directly exposed to the pollutants. Thus, indirect exposure to microplastics and pesticides through diet can potentially have bottom-up effects on the trophic webs. This result supports our fifth prediction and is the first study to our knowledge to show these effects. Our experimental design is somewhat artificial because in nature all three levels might be affected by DMT exposure, for example through run-off processes caused by heavy rains. We did however use the aforementioned design because we wanted to study the effect of transfers from one level to another per se, i.e., study the effect of prey exposed to MPs and DMT on the predator microbiome, instead of assessing the direct exposure on the whole system. A more optimal design that requires future investigation would be to run another experiment also applying the DMT at the level of the top predator. The treatment with MPs only on *Daphnia* is realistic because MPs are probably only ingested directly by the filter-feeding *Daphnia* and only indirectly in the second and third order predators (damselfly and dragonfly respectively).

There are plenty of studies showing that diet, in terms of which species are consumed, influences the microbiome [5, 12, 92–96]. In this study, we instead showed that the microbiome of a predator is influenced by the environment experienced by its prey (our treatments). We acknowledge that we do not have evidence that the microplastics are physically transferred to higher trophic levels. Thus, we do not know whether the effect on higher trophic levels is a direct effect from the exposure to microplastics, or if it is an indirect effect by the prey. There are two ways that a predator could be indirectly affected by the prey exposed to microplastics. First, the disturbed microbiome of the prey may be carried over and colonize the predator, similar to the effects of ingesting probiotics [97, 98]. Second, the microbiome has been shown to affect the metabolism of the host [99–103] by producing metabolites that affect host physiology [102–105], which in turn could affect a predator’s microbiome.

## Conclusions

In general, the organisms on the different trophic levels harbor a diverse microbial community, and the host–microbiome differed from the microbiome in the environment. Our results showed that the exposure to pesticides and microplastics at lower trophic levels had an effect on the microbiome of organisms at higher trophic levels, and whether this was caused by direct effects of pesticide/microplastic transfer or by indirect effects carried over via predation remains to be investigated. It is possible that MPs in combination with other pollutants can affect non-target organisms and their microbiome and be translocated from aquatic to soil environments via metamorphic organisms. We suggest further experimentation on tracking MPs and its interactions with the host–microbiome. For example, metatranscriptomics and metabolic variation of functions on the microbial communities could be tested under DMT and MPs exposure.

## Supplementary Information

**Additional file1** (CSV 25 KB) The unprocessed data that was used to perform the predation analysis over time. The damselfly larvae were exposed to the following treatment: microplastics, deltamethrin pesticide, deltamethrin & microplastics, and control. The data showed damselfly survival counts (Survival), dragonfly predation counts (Predation), predation times in minutes (Time), dragonfly IDs (Number), categorical predation times (TimeC), categorical dragonfly IDs (NumberC), damselfly survival percentages (SurvivalP), microplastics as a binomial variable (MP), deltamethrin as a binomial variable (Delta), and the damselflies' survival (SurvivalDam1, SurvivalDam2, and SurvivalDam2).

**Additional** **file2**. **Table S1**: Results for the MANOVA testing the effects of exposure to microplastics (MPs) and deltamethrin (DMT) on the relative abundance of the six main microbiota phyla in the Daphnia. The univariate models testing the effects of exposure to MPs and DMT on the relative abundance of the six main microbiota phyla are also included. Significant and marginally non-significant *p*-values are highlighted in bold. **Table S2**: Results for the MANOVA testing the effects of exposure to microplastics (MPs) and deltamethrin (DMT) on the relative abundance of the six main microbiota genera (g__) or microbiota families (f__), if genus was not possible to be assigned in the Daphnia microbiome. The univariate models testing the effects of exposure to MPs and DMT on the relative abundance of the six main microbiota phyla are also included. Significant and marginally non-significant *p*-values are highlighted in bold. **Table S3**: Results for the univariate models testing the effects of exposure to microplastics (MPs) and deltamethrin (DMT) on the relative abundance on low abundant genera (g__) or families (f__), if genus was not possible to be assigned, of the main phyla that constitute more than 0.5% of the total relative abundance in the Daphnia microbiome. Significant and marginally non-significant *p*-values are highlighted in bold. **Table S4**: Results for the MANOVA testing the effects of exposure to microplastics (MPs) and deltamethrin (DMT) on the relative abundance of the six main microbiota phyla in the Damselfly. The univariate models testing the effects of exposure to MPs and DMT on the relative abundance of the six main microbiota phyla are also included. Significant and marginally non-significant *p*-values are highlighted in bold. **Table S5**: Results for the MANOVA testing the effects of exposure to microplastics (MPs) and deltamethrin (DMT) on the relative abundance of the six main microbiota genera (g__) or microbiota families (f__), if genus was not possible to be assigned in the Damselfly microbiome. The univariate models testing the effects of exposure to MPs and DMT on the relative abundance of the six main microbiota phyla are also included. Significant and marginally non-significant *p*-values are highlighted in bold. **Table S6**: Post hoc contrasts on the univariate relative abundances of the main six phyla of the microbiome of Daphnia, damselflies and dragonflies, testing differences between treatments: Control, exposure to microplastics (MPs), exposure to deltamethrin (DMT), and the combined exposure to MPs and DMT. Only significant and marginally non-significant *p*-values are shown. **Table S7**: Results for the univariate models testing the effects of exposure to microplastics (MPs) and deltamethrin (DMT) on the relative abundance on low abundant genera (g__) or families (f__), if genus was not possible to be assigned, of the main phyla that constitute more than 0.5% of the total relative abundance in the Damselfly microbiome. Significant and marginally non-significant *p*-values are highlighted in bold. **Table S8**: Results for the MANOVA testing the effects of exposure to microplastics (MPs) and deltamethrin (DMT) on the relative abundance of the six main microbiota phyla in the Dragonfly. The univariate models testing the effects of exposure to MPs and DMT on the relative abundance of the six main microbiota phyla are also included. Significant and marginally non-significant *p*-values are highlighted in bold. **Table S9**: Results for the MANOVA testing the effects of exposure to microplastics (MPs) and deltamethrin (DMT) on the relative abundance of the six main microbiota genera (g__) or microbiota families (f__), if genus was not possible to be assigned in the Dragonfly microbiome. The univariate models testing the effects of exposure to MPs and DMT on the relative abundance of the six main microbiota phyla are also included. Significant and marginally non-significant *p*-values are highlighted in bold. **Figure S1**: Class level taxa relative abundance of the microbiome of Daphnia, the damselfly and the dragonfly, including the relative abundance of the water microbiota extracted from the filters. The exposure treatments were: microplastics (MPs), the pesticide deltamethrin (DMT), a combination of MPs and DMT, and the Control group (no exposure to either MPs or DMT). **Table S10**: Results for the univariate models testing the effects of exposure to microplastics (MPs) and deltamethrin (DMT) on the relative abundance on low abundant genera (g__) or microbial taxonomic rank, if genus was not possible to be assigned, of the main phyla that constitute more than 0.5% of the total relative abundance in the Dragonfly microbiome. Significant and marginally non-significant *p*-values are highlighted in bold. **Table S11**: Results of the GLMM testing the effects of exposure to microplastics (MPs), deltamethrin (DMT) and their interaction on damselfly survival. Significant and marginally nonsignificant *p*-values are highlighted in bold.

## Data Availability

Data is available on request from Javier Edo Varg at jedovarg@gmail.com. Data have been uploaded to the European nucleotide archive (ENA) and it will be available after publication. With accession number: PRJEB45338 and the study unique name: ena-STUDY-UPPSALA UNIVERISTY-28-05-2021-04:43:07:156-465.
