# Supplementary material for: Microplastic exposure across trophic levels: effects on the host–microbiota of freshwater organisms
Source: Environ Microbiome. 2022 Jul 6;17:36. doi: 10.1186/s40793-022-00429-x (PMC9258161; doi:10.1186/s40793-022-00429-x)
Supplement: Supplementary file 2 — Additional file2. Table S1: Results for the MANOVA testing the effects of exposure to microplastics (MPs) and deltamethrin (DMT) on the relative abundance of the six main microbiota phyla in the Daphnia. The univariate models testing the effects of exposure to MPs and DMT on the relative abundance of the six main microbiota phyla are also included. Significant and marginally non-significant p-values are highlighted in bold. Table S2: Results for the MANOVA testing the effects of exposure to microplastics (MPs) and deltamethrin (DMT) on the relative abundance of the six main microbiota genera (g__) or microbiota families (f__), if genus was not possible to be assigned in the Daphnia microbiome. The univariate models testing the effects of exposure to MPs and DMT on the relative abundance of the six main microbiota phyla are also included. Significant and marginally non-significant p-values are highlighted in bold. Table S3: Results for the univariate models testing the effects of exposure to microplastics (MPs) and deltamethrin (DMT) on the relative abundance on low abundant genera (g__) or families (f__), if genus was not possible to be assigned, of the main phyla that constitute more than 0.5% of the total relative abundance in the Daphnia microbiome. Significant and marginally non-significant p-values are highlighted in bold. Table S4: Results for the MANOVA testing the effects of exposure to microplastics (MPs) and deltamethrin (DMT) on the relative abundance of the six main microbiota phyla in the Damselfly. The univariate models testing the effects of exposure to MPs and DMT on the relative abundance of the six main microbiota phyla are also included. Significant and marginally non-significant p-values are highlighted in bold. Table S5: Results for the MANOVA testing the effects of exposure to microplastics (MPs) and deltamethrin (DMT) on the relative abundance of the six main microbiota genera (g__) or microbiota families (f__), if genus was not possible to be [file 40793_2022_429_MOESM2_ESM.pdf]

Supplementary Materials for:

# Microplastic exposure across trophic levels: effects on the host-microbiota of freshwater organisms

Javier Edo Varg, David Outomuro, Warren Kunce, Lukas Kuehrer, Richard Svanbäck, and Frank Johansson

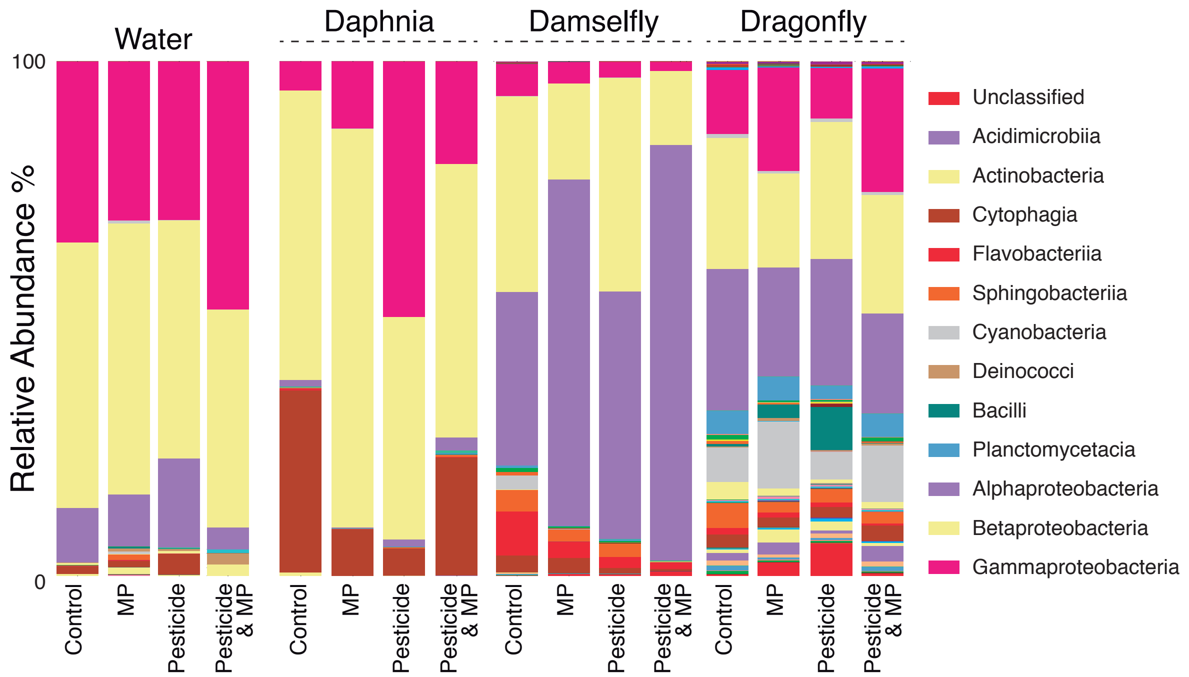


**Figure S1**. Class level taxa relative abundance of the microbiome of *Daphnia*, the damselfly and the dragonfly, including the relative abundance of the water microbiota extracted from the filters. The exposure treatments were: microplastics (MPs), the pesticide deltamethrin (DMT), a combination of MPs and DMT, and the Control group (no exposure to either MPs or DMT).

Table S1. Results for the MANOVA testing the effects of exposure to microplastics (MPs) and deltamethrin (DMT) on the relative abundance of the six main microbiota phyla in the *Daphnia*. The univariate models testing the effects of exposure to MPs and DMT on the relative abundance of the six main microbiota phyla are also included. Significant and marginally non-significant p-values are highlighted in bold.

| Organism | Variable | MPs | DMT | MPs:DMT |
|---|---|---|---|---|
| | **MANOVA** | | | |
| | Pillai | 0.413 | 0.449 | 0.447 |
| | Approx $F_{6,8}$ | 0.937 | 1.088 | 1.079 |
| | p-value | 0.518 | 0.443 | 0.447 |
| | **Proteobacteria** | | | |
| | $\chi^2_{1,13}$ | 5.286 | 4.239 | 5.468 |
| | p-value | **0.021** | **0.039** | **0.019** |
| | **Bacteroidetes** | | | |
| ***Daphnia*** | $\chi^2_{1,13}$ | 7.399 | 5.81 | 8.009 |
| | p-value | **0.006** | **0.016** | **0.005** |
| | **Actinobacteria** | | | |
| | $\chi^2_{1,13}$ | 6.519 | 4.738 | 5.403 |
| | p-value | **0.011** | **0.029** | **0.02** |
| | **Planctomycetes** | | | |
| | $\chi^2_{1,13}$ | 0.075 | 3.997 | 0.101 |
| | p-value | 0.784 | **0.046** | 0.75 |
| | **Firmicutes** | | | |
| | $\chi^2_{1,13}$ | 0.399 | 0.879 | 1.154 |
| | p-value | 0.528 | 0.348 | 0.283 |
| | **Cyanobacteria** | | | |
| | $\chi^2_{1,13}$ | 0.049 | 0.002 | 0.476 |
| | p-value | 0.824 | 0.963 | 0.49 |

Table S2. Results for the MANOVA testing the effects of exposure to microplastics (MPs) and deltamethrin (DMT) on the relative abundance of the six main microbiota genera (g__) or microbiota families (f__), if genus was not possible to be assigned in the *Daphnia* microbiome. The univariate models testing the effects of exposure to MPs and DMT on the relative abundance of the six main microbiota phyla are also included. Significant and marginally non-significant p-values are highlighted in bold.

| Organism | Variable | MPs | DMT | MPs:DMT |
|---|---|---|---|---|
| | **MANOVA** | | | |
| | Pillai | 0.338 | 0.561 | 0.637 |
| | Approx $F_{6,8}$ | 0.682 | 1.705 | 2.337 |
| | p-value | 0.670 | 0.237 | 0.132 |
| | **f__*Comamonadaceae*** | | | |
| | $\chi^2_{1,13}$ | 0.664 | 0.012 | 0.186 |
| | p-value | 0.415 | 0.914 | 0.666 |
| | **g__*Leadbetterella*** | | | |
| ***Daphnia*** | $\chi^2_{1,13}$ | 7.159 | 5.612 | 7.746 |
| | p-value | **0.007** | **0.018** | **0.005** |
| | **g__*Aeromonas*** | | | |
| | $\chi^2_{1,13}$ | 2.210 | 4.934 | 5.07 |
| | p-value | 0.137 | **0.0263** | **0.024** |
| | **g__*Pseudomonas*** | | | |
| | $\chi^2_{1,13}$ | 0.054 | 0.595 | 1.134 |
| | p-value | 0.815 | 0.44 | 0.287 |
| | **g__*Curvibacter*** | | | |
| | $\chi^2_{1,13}$ | 3.769 | 3.014 | 0.733 |
| | p-value | **0.052** | **0.083** | 0.392 |
| | **g__*Limnobacter*** | | | |
| | $\chi^2_{1,13}$ | 5.596 | 3.099 | 3.224 |
| | p-value | **0.018** | **0.078** | **0.072** |

Table S3. Results for the univariate models testing the effects of exposure to microplastics (MPs) and deltamethrin (DMT) on the relative abundance on low abundant genera (g__) or families (f__), if genus was not possible to be assigned, of the main phyla that constitute more than 0.5% of the total relative abundance in the *Daphnia* microbiome. Significant and marginally non-significant p-values are highlighted in bold.

| **Organism** | **Phyla** | **Variable** | **MPs** | **DMT** | **MPs:DMT** |
|---|---|---|---|---|---|
| *Daphnia* | **Bacteroidetes** | **f__*Sphingomonadaceae*** | | | |
| | | $\chi^2_{1,13}$ | 8.793 | 2.912 | 4.849 |
| | | p-value | **0.003** | **0.088** | **0.028** |
| | | **g__*Acidovorax*** | | | |
| | | $\chi^2_{1,13}$ | 3.069 | 2.316 | 0.364 |
| | | p-value | **0.08** | 0.128 | 0.546 |
| | | **o__*Burkholderiales*** | | | |
| | | $\chi^2_{1,13}$ | 4.736 | 2.812 | 4.425 |
| | | p-value | **0.029** | **0.094** | **0.035** |
| | **Actinobacteria** | **f__*Microbacteriaceae*** | | | |
| | | $\chi^2_{1,13}$ | 3.314 | 3.510 | <0.001 |
| | | p-value | **0.069** | **0.061** | 0.999 |
| | | **g__*Salinibacterium*** | | | |
| | | $\chi^2_{1,13}$ | 3.672 | 3.135 | <0.001 |
| | | p-value | **0.055** | **0.077** | 0.999 |
| | | **g__*Microbacterium*** | | | |
| | | $\chi^2_{1,13}$ | 6.721 | 7.185 | <0.001 |
| | | p-value | **0.009** | **0.007** | 0.999 |
| | **Planctomycetes** | **g__*Pirellula*** | | | |
| | | $\chi^2_{1,13}$ | 0.031 | 1.921 | 0.132 |
| | | p-value | 0.861 | 0.166 | 0.716 |
| | | **g__*Planctomyces*** | | | |
| | | $\chi^2_{1,13}$ | 0.103 | 5.206 | 0.092 |
| | | p-value | 0.748 | **0.022** | 0.762 |
| | | **Unclassified** | | | |
| | | $\chi^2_{1,13}$ | 0.011 | 0.635 | 0.594 |
| | | p-value | 0.916 | 0.425 | 0.441 |

Table S4. Results for the MANOVA testing the effects of exposure to microplastics (MPs) and deltamethrin (DMT) on the relative abundance of the six main microbiota phyla in the Damselfly. The univariate models testing the effects of exposure to MPs and DMT on the relative abundance of the six main microbiota phyla are also included. Significant and marginally non-significant p-values are highlighted in bold.

| Organism | Variable | MPs | DMT | MPs:DMT |
|---|---|---|---|---|
| Damselfly | **MANOVA** | | | |
| | Pillai | 0.479 | 0.341 | 0.202 |
| | Approx $F_{6,15}$ | 2.303 | 1.292 | 0.634 |
| | p-value | **0.089** | 0.319 | 0.701 |
| | **Proteobacteria** | | | |
| | $\chi^2_{1,20}$ | 5.161 | 3.301 | 0.458 |
| | p-value | **0.023** | **0.069** | 0.499 |
| | **Bacteroidetes** | | | |
| | $\chi^2_{1,20}$ | 2.07 | 1.519 | 0.332 |
| | p-value | 0.15 | 0.218 | 0.564 |
| | **Cyanobacteria** | | | |
| | $\chi^2_{1,20}$ | 7.343 | 3.851 | 0.164 |
| | p-value | **0.007** | **0.050** | 0.685 |
| | **Planctomycetes** | | | |
| | $\chi^2_{1,20}$ | 5.341 | 1.746 | 0.104 |
| | p-value | **0.021** | 0.186 | 0.747 |
| | **Unclassified** | | | |
| | $\chi^2_{1,20}$ | 8.057 | 2.208 | 0.216 |
| | p-value | **0.004** | 0.137 | 0.642 |
| | **Gemmatimonadetes** | | | |
| | $\chi^2_{1,20}$ | 4.413 | 1.674 | 0.09 |
| | p-value | **0.036** | 0.196 | 0.764 |

Table S5. Results for the MANOVA testing the effects of exposure to microplastics (MPs) and deltamethrin (DMT) on the relative abundance of the six main microbiota genera (g__) or microbiota families (f__), if genus was not possible to be assigned in the Damselfly microbiome. The univariate models testing the effects of exposure to MPs and DMT on the relative abundance of the six main microbiota phyla are also included. Significant and marginally non-significant p-values are highlighted in bold.

| Organism | Variable | MPs | DMT | MPs:DMT |
|---|---|---|---|---|
| Damselfly | **MANOVA** | | | |
| | Pillai | 0.65 | 0.291 | 0.454 |
| | Approx $F_{6,15}$ | 4.641 | 1.024 | 2.081 |
| | p-value | **0.007** | 0.447 | 0.117 |
| | **g__*Rickettsia*** | | | |
| | $\chi^2_{1, 20}$ | 15.214 | 3.204 | 1.513 |
| | p-value | **<0.001** | **0.073** | 0.219 |
| | **f__*Comamonadaceae*** | | | |
| | $\chi^2_{1, 20}$ | 0.989 | 0.098 | <0.001 |
| | p-value | 0.320 | 0.754 | 0.976 |
| | **g__*Flavobacterium*** | | | |
| | $\chi^2_{1, 20}$ | 5.833 | 4.644 | 0.223 |
| | p-value | **0.016** | **0.031** | 0.637 |
| | **g__*Methylophilus*** | | | |
| | $\chi^2_{1, 20}$ | 0.013 | 0.014 | 3.427 |
| | p-value | 0.914 | 0.907 | **0.064** |
| | **f__*Sphingomonadaceae*** | | | |
| | $\chi^2_{1, 20}$ | 5.823 | 2.474 | 0.546 |
| | p-value | **0.0158** | 0.116 | 0.46 |
| | **g__*Rhizobacter*** | | | |
| | $\chi^2_{1, 20}$ | 10.263 | 1.593 | 0.163 |
| | p-value | **<0.001** | 0.207 | 0.686 |

Table S6. Post hoc contrasts on the univariate relative abundances of the main six phyla of the microbiome of *Daphnia*, damselflies and dragonflies, testing differences between treatments: Control, exposure to microplastics (MPs), exposure to deltamethrin (DMT), and the combined exposure to MPs and DMT.  Only significant and marginally non-significant p-values are shown.

| | **Variable** | **contrast** | **p-value** |
|---|---|---|---|
| *Daphnia* | **Proteobacteria** | Control – MPs | 0.098 |
| | **Bacteroidetes** | Control – MPs | 0.075 |
| Damselfly | **Proteobacteria** | Control – MPs & DMT | 0.044 |
| | **Unclassified** | Control – MPs | 0.047 |
| | | Control – MPs & DMT | 0.001 |
| Dragonfly | **Bacteroidetes** | Control – MPs | 0.036 |
| | **Actinobacteria** | Control – MPs | 0.045 |

Table S7. Results for the univariate models testing the effects of exposure to microplastics (MPs) and deltamethrin (DMT) on the relative abundance on low abundant genera (g__) or families (f__), if genus was not possible to be assigned, of the main phyla that constitute more than 0.5% of the total relative abundance in the Damselfly microbiome. Significant and marginally non-significant p-values are highlighted in bold.

| Organism | Phyla | Variable | MPs | DMT | MPs:DMT |
|---|---|---|---|---|---|
| Damselfly | Bacteroidetes | **g__*Sediminibacterium*** | | | |
| | | $\chi^2_{1,13}$ | 0.006 | 0.222 | 2.523 |
| | | p-value | 0.939 | 0.637 | 0.112 |
| | | **g__*Leadbetterella*** | | | |
| | | $\chi^2_{1,13}$ | 0.474 | 0.011 | 1.279 |
| | | p-value | 0.491 | 0.917 | 0.258 |
| | | **g__*Emticicia*** | | | |
| | | $\chi^2_{1,13}$ | 0.419 | 2.620 | 0.134 |
| | | p-value | 0.517 | 0.105 | 0.714 |
| | Cyanobacteria | **g__*Leptolyngbya*** | | | |
| | | $\chi^2_{1,13}$ | 7.414 | 3.876 | 0.015 |
| | | p-value | **0.006** | **0.049** | 0.902 |
| | | **f__*Sphingomonadaceae*** | | | |
| | | $\chi^2_{1,13}$ | 5.823 | 2.474 | 0.546 |
| | | p-value | **0.016** | 0.116 | 0.46 |
| | | **Unclassified** | | | |
| | | $\chi^2_{1,13}$ | 8.63 | 2.412 | 0.293 |
| | | p-value | **0.003** | 0.120 | 0.588 |

Table S8. Results for the MANOVA testing the effects of exposure to microplastics (MPs) and deltamethrin (DMT) on the relative abundance of the six main microbiota phyla in the Dragonfly. The univariate models testing the effects of exposure to MPs and DMT on the relative abundance of the six main microbiota phyla are also included. Significant and marginally non-significant p-values are highlighted in bold

| Organism | Variable | MPs | DMT | MPs:DMT |
|---|---|---|---|---|
| Dragonfly | **MANOVA** | | | |
| | Pillai | 0.39 | 0.084 | 0.302 |
| | Approx $F_{6,27}$ | 2.877 | 0.411 | 1.949 |
| | p-value | **0.027** | 0.865 | 0.109 |
| | **Proteobacteria** | | | |
| | $\chi^2_{1, 32}$ | 0.865 | 0.811 | 1.116 |
| | p-value | 0.352 | 0.368 | 0.291 |
| | **Cyanobacteria** | | | |
| | $\chi^2_{1, 32}$ | 0.431 | 1.293 | 0.742 |
| | p-value | 0.511 | 0.255 | 0.389 |
| | **Bacteroidetes** | | | |
| | $\chi^2_{1, 32}$ | 8.057 | 2.644 | 3.018 |
| | p-value | **0.004** | 0.104 | **0.082** |
| | **Planctomycetes** | | | |
| | $\chi^2_{1, 32}$ | 1.125 | 3.868 | 3.955 |
| | p-value | 0.289 | **0.049** | **0.047** |
| | **Actinobacteria** | | | |
| | $\chi^2_{1, 32}$ | 7.438 | 0.003 | 0.129 |
| | p-value | **0.006** | 0.957 | 0.72 |
| | **Acidobacteria** | | | |
| | $\chi^2_{1, 32}$ | 4.07 | 2.288 | 3.207 |
| | p-value | **0.044** | 0.13 | **0.073** |

Table S9. Results for the MANOVA testing the effects of exposure to microplastics (MPs) and deltamethrin (DMT) on the relative abundance of the six main microbiota genera (g__) or microbiota families (f__), if genus was not possible to be assigned in the Dragonfly microbiome. The univariate models testing the effects of exposure to MPs and DMT on the relative abundance of the six main microbiota phyla are also included. Significant and marginally non-significant p-values are highlighted in bold

| Organism | Variable | MPs | DMT | MPs:DMT |
|---|---|---|---|---|
| Dragonfly | **MANOVA** | | | |
| | Pillai | 0.367 | 0.119 | 0.324 |
| | Approx $F_{6,27}$ | 2.609 | 0.612 | 2.153 |
| | p-value | **0.04** | 0.719 | **0.08** |
| | **f__*Comamonadaceae*** | | | |
| | $\chi^2_{1,32}$ | 12.227 | 5.470 | 7.028 |
| | p-value | **<0.001** | **0.019** | **0.008** |
| | **g__*Ralstonia*** | | | |
| | $\chi^2_{1,32}$ | 3.199 | 3.623 | 2.451 |
| | p-value | **0.074** | **0.057** | 0.117 |
| | **g__*Chamaesiphon*** | | | |
| | $\chi^2_{1,32}$ | 3.640 | 0.043 | 0.073 |
| | p-value | **0.056** | 0.835 | 0.787 |
| | **g__*Aeromonas*** | | | |
| | $\chi^2_{1,32}$ | 0.058 | 0.462 | 3.851 |
| | p-value | 0.809 | 0.497 | **0.05** |
| | **g__*Rhodobacter*** | | | |
| | $\chi^2_{1,32}$ | 10.236 | 0.003 | 0.129 |
| | p-value | **0.006** | 0.957 | 0.72 |
| | **g__*Acinetobacter*** | | | |
| | $\chi^2_{1,32}$ | 4.07 | 2.048 | 1.6 |
| | p-value | **0.001** | 0.152 | 0.207 |

Table S10. Results for the univariate models testing the effects of exposure to microplastics (MPs) and deltamethrin (DMT) on the relative abundance on low abundant genera (g__) or microbial taxonomic rank, if genus was not possible to be assigned, of the main phyla that constitute more than 0.5% of the total relative abundance in the Dragonfly microbiome. Significant and marginally non-significant p-values are highlighted in bold.

| Organism | Phyla | Variable | MPs | DMT | MPs:DMT |
|---|---|---|---|---|---|
| Dragonfly | Cyanobacteria | **c__Chloroplast** | | | |
| | | $\chi^2_{1,13}$ | 7.281 | 7.106 | 3.574 |
| | | p-value | **0.007** | **0.008** | **0.06** |
| | | **f__*FamilyI*** | | | |
| | | $\chi^2_{1,13}$ | 0.652 | 4.026 | 3.376 |
| | | p-value | 0.419 | **0.045** | **0.066** |
| | | **g__*Leptolyngbya*** | | | |
| | | $\chi^2_{1,13}$ | 0.096 | 2.003 | 0.012 |
| | | p-value | 0.757 | 0.157 | 0.912 |
| | Bacteroidetes | **f__*Saprospiraceae*** | | | |
| | | $\chi^2_{1,13}$ | 8.781 | 3.033 | 1.691 |
| | | p-value | **0.003** | **0.082** | 0.193 |
| | | **g__*Emticicia*** | | | |
| | | $\chi^2_{1,13}$ | 4.054 | 0.004 | 3.229 |
| | | p-value | **0.044** | 0.948 | **0.072** |
| | | **Unclassified** | | | |
| | | $\chi^2_{1,13}$ | 5.902 | 15.090 | 13.292 |
| | | p-value | **0.015** | **<0.001** | **<0.001** |
| | Planctomycetes | **g__*Pirellula*** | | | |
| | | $\chi^2_{1,13}$ | <0.001 | 3.8 | 1.798 |
| | | p-value | 0.993 | **0.051** | 0.18 |
| | | **g__*Planctomyces*** | | | |
| | | $\chi^2_{1,13}$ | 5.077 | 3.376 | 6.532 |
| | | p-value | **0.024** | **0.066** | **0.011** |
| | | **g__*Gemmata*** | | | |
| | | $\chi^2_{1,13}$ | 0.382 | 1.322 | 0.038 |
| | | p-value | 0.536 | 0.250 | 0.845 |

Table S11. Results of the GLMM testing the effects of exposure to microplastics (MPs), deltamethrin (DMT) and their interaction on damselfly survival. Significant and marginally non-significant p-values are highlighted in bold.

| **Survival** | **MPs** | **DMT** | **Time** | **MPs:DMT** | **MPs:Time** | **DMT:Time** | **MPs:DMT:Time** |
|---|---|---|---|---|---|---|---|
| **Damselfly** | | | | | | | |
| $F_{1, 532}$ | 0.363 | 7.987 | 212.814 | 0.019 | 0.4433 | 2.353 | 0.654 |
| p-value | 0.547 | **0.005** | **<0.001** | 0.889 | 0.506 | 0.126 | 0.419 |
